# Electrocatalytic Performance of Chemically Synthesized PIn-Au-SGO Composite toward Mediated Biofuel Cell Anode

**DOI:** 10.1038/s41598-017-13539-1

**Published:** 2017-10-17

**Authors:** Ruma Perveen, Inamuddin, Sufia ul Haque, Abu Nasar, Abdullah M. Asiri, Ghulam Md Ashraf

**Affiliations:** 1 Advanced Functional Materials Laboratory, Department of Applied Chemistry, Faculty of Engineering and Technology, Aligarh Muslim University, Aligarh, 202002 India; 2 Chemistry Department, Faculty of Science, King Abdulaziz University, Jeddah, 21589 Saudi Arabia; 3 Centre of Excellence for Advanced Materials Research (CEAMR), King Abdulaziz University, P. O. Box 80203, Jeddah, 21589 Saudi Arabia; 4 King Fahd Medical Research Center, King Abdulaziz University, Jeddah, 21589 Saudi Arabia

## Abstract

The proposed work intended to make an intellectual contribution to the domain of green nanotechnology which emphasizes the chemical synthesis of a conducting nanocomposite based on the incorporation of gold nanoparticles (Au) into the redox matrix of polyindole (PIn) along with the subsequent improvement in the overall properties of the composite by the addition of sulfonated graphene oxide (SGO). The bioanode was developed by the deposition of the PIn-Au-SGO nanocomposite with subsequent immobilization of ferritin (Frt) and glucose oxidase (GOx) on the glassy carbon electrode (GC). The successful application of the PIn-Au-SGO nanocomposite toward the development of a ferritin-mediated glucose biofuel cell anode was studied by the electrochemical characterization of the constructed bioanode (GC-PIn-Au-SGO/Frt/GOx) for the bioelectrocatalytic oxidation of glucose. The maximum current density obtained by the modified bioanode was found to be 17.8 mA cm^−2^ at the limiting glucose concentration of 50 mM in 0.1 M K_4_Fe(CN)_6_ at a scan rate of 100 mVs^−1^. The lifetime of the concerned bioelectrode when stored at 4 °C was estimated to be 53 days approximately. The appreciable results of the structural and electrochemical characterization of the PIn-Au-SGO based bioelectrode reveal its potential applications exclusively in implantable medical devices.

## Introduction

In the era of nanoscience and nanotechnology, with both theoretical significance and practical applications, nanostructured hybrid systems based on metal or semiconductor nanoparticles and organic compounds have fueled extensive research efforts^1–6^ to explore their concealed dimensions. The interest toward the development of new and impactful hybrid materials is increasing rapidly for a wide domain of applications including but not limited to biomedical technologies, sensors, catalysis, functional materials and electronics^7–12^. Efforts to create miniaturized devices are fundamentally inspired by the unambiguous, simple, cost effective and significantly feasible synthesis of redox active conductive hybrid nanocomposites. Especially, the coordination of metallic (e.g. Au, Pt, Pd, or Cu) or semiconductor nanoparticles (CdS, CdSe, CdTe, or TiO_2_) into conductive redox polymers, for example, polypyrrole (PPy), polyaniline (PAni), polycarbazole (PC), polyindole (PIn) and so forth is the state-of the-art technique owing to the strong electronic interactions between the nanoparticles and the polymer grids. Reports till now, have put in plain view that the electrocatalytic properties of nanoparticles are enhanced by the conductive condition given by the polymeric frameworks^13^, while the electronic conductivity of the composite frameworks is raised within the sight of metal nanoparticles installed into the polymeric structure.

Heterocyclic conducting polymers like PAni, PPy, PC and their substituted derivatives^14,15^ have attracted significant enthusiasm because of their potential industrial applications. These conducting polymers fill in as a conducting leading framework for the scattering of nanometallics because of their great stability and support of the heteroatom during redox reaction and in addition capacity to hold nanoclusters inside the network^16,17^. Among heterocyclic conducting polymers, PIn is a potential contender for its dynamic properties rising as an outcome of the combinational property of both poly(p-phenylene) and polypyrrole in concert^18^ in areas including electronic hardware, electrocatalysis, energy, and pharmacology. In its doped state, PIn is green, with an electrical conductivity in the scope of 5 × 10^−4^– 8 × 10^−2^ S cm^−1^ ^19^. PIn can be effortlessly prepared either electrochemical or by synthetic oxidation of indole monomer (C_8_NH_7_) with genuinely good thermal stability, high redox activity, moderate degradation rate, and better air stability in contrast with PAni and PPy^20,21^. Different literary works have been published uncovering the electrochemical properties of PIn, utilized in battery anodes, corrosion protection, nano light-emitting devices^7,22–24^ etc. Among different nanometallics, nano-gold bunches have pulled in extensive consideration over the current years as a result of the predominant practical properties which is empowering them to be relevant in different fields extending from catalysis to electronic applications and sensors^25–28^. A few nanocomposites of gold with an extensive variety of heterocyclic electroactive polymers, for example, PPy, PAni, PTh, PC, and PIn^29–33^ have been produced by using synthetic and electrochemical courses wherein gold goes about as a promoter for different applications, for example, catalysis, sensor, memory gadgets, and electronic applications due to a synergic impact between constituents of the nanocomposite^34–41^. As of late, a composite of nanosized gold and PIn was prepared by means of *in situ* polymerization of indole, highlighting its nanoscale electrical properties^42^. Our commitment towards the green nanotechnology upset is to orchestrate a novel electron conducting nanocomposite and decide its electrocatalytic oxidation for a glucose based biofuel cell by manufacturing a bioanode. In this work, a novel nanocomposite of PIn, gold, and sulfonated graphene oxide (SGO) is chemically synthesized. Sulfonated graphene oxide (SGO) and its subordinate composites possess an extensive variety of utilizations, for example, in supercapacitors, biosensors and biofuel cells. Functionalization of graphene oxide with sulphonic assemble enhances thermal stability and compatibility with different substrates^43–45^. Sulfonate group is an essential functional group as it can dissociate the mobile proton for ionic conductivity with the assistance of the solvation effect^46^. The consolidation of sulfonated graphene oxide into the polymer-metal composite may increment the overall electrical and electrochemical properties of the whole composite because of the graphitic structure of the GO and sulphonic groups joined to GO. Recently, a composite film of SGO and PPy was electrochemically deposited from the aqueous solution of pyrrole monomer with a massive change in the conductivity and electrochemical properties of the SGO-PPy composite films compared to that of PPY films alone^47^. A composite of sulfonated graphene-supported vertically aligned nanorods has been utilized for the manufacture of high-performance supercapacitors^48^. The SG-SiO_2_/Nafion proton exchange membranes (PEMs) with improved selectivity were obtained to use in direct methanol fuel cells^49^.

The major concern of depleting natural energy resources due to excessive exploitation of fuels leaned towards the emergence of alternative renewable energy sources for future generations. Tremendous work has been done in this very field of fuel cells and biofuel cells which are entitled as future energy resources. Fundamentally, a fuel cell is an electrochemical device that drives out electrical power by the transformation of chemical potential of a fuel and an oxidant. The fuel cells which employ biological catalysts (enzymes or microorganisms) collectively feature the well-renowned biofuel cells^30,50^.

For as long as a couple of decades enzymatic glucose biofuel cells (EBFCs) which change over substance energy of a fuel (glucose) into electrical energy through electrochemical responses including biochemical pathways, have been the predominant technology^51^. The working principle of EBFCs is the same as in conventional fuel cells, wherein the electrons are generated during redox processes and the transfer of these electrons from the anode to cathode is driven through an external electrical circuit, causing power generation. These biofuel cells are not only a means of biomass conversion but also fill the need of an alternative source of energy for implantable biomedical gadgets, for example, transmitters, scaled down sensors, pacemaker and manufactured organs^52–54^.

**Figure 1:**
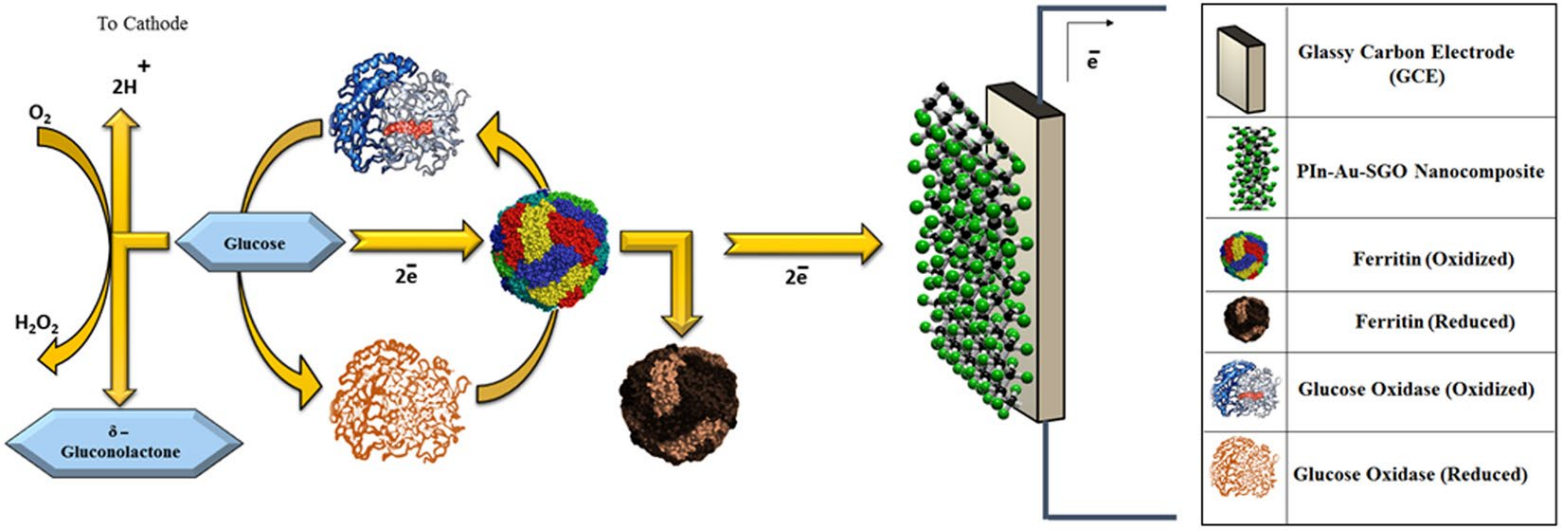
Schematic representation of this research work.

Enzymatic glucose biofuel cells, utilizing enzymes as electrocatalysts, are advantageous from specificity for oxygen reduction and glucose oxidation reactions. However, low stacking and poor stability of enzyme that prompts low power density and short lifetime restrict them. The execution of a biofuel cell is dominatingly represented by the choice of anode and cathode architectures^55–58^. This study collectively reports a fast, simple, proficient and cost effective preparation of PIn-Au-SGO nanocomposite for its electrocatalytic execution as a bioanode for enzymatic glucose biofuel cells. Figure 1 shows the schematic representation of this research work.

## Materials and Methods

### Materials

Indole (Distilled), hydrochloric acid and potassium ferrocyanide [K_4_Fe(CN)_6_] were purchased from Merck, India. Chloroauric acid (HAuCl_4_) was gotten from SRL, Sisco India Ltd. KMnO_4_, H_2_SO_4_ and dimethyl formamide (DMF) were purchased from Central Drug House, India. Glucose oxidase (GOx) (Activity 100,000–150,000 units g^−1^ protein) from *Aspergillus niger*, the ferritin (10 mg mL^−1^ in 0.15 M NaCl) from stallion spleen, glutaraldehyde and graphene utilized were acquired from Sigma-Aldrich Chemicals, India. Phosphate buffers of pH 5.0 and 7.0 (Otto Pvt. Ltd. India), and D-(+)-glucose anhydrous (Himedia Laboratories Pvt. Ltd. India) were utilized as gotten. All other reagents utilized were of analytical grade. Distilled water was utilized all through the tests.

### Synthesis of sulfonated graphene oxide (SGO)

#### Graphene oxide synthesis

Graphene oxide was prepared by a modified Hummers strategy^59^ including both oxidation and peeling of graphite sheets utilizing KMnO_4_ and H_2_SO_4_ as an oxidizing agent. The graphite pieces (2.0 g) were dissolved into 50 mL of concentrated H_2_SO_4_ in a 1 L volumetric jar and after that, the mixture was consistently unsettled for 1 h inside an ice bath using a magnetic stirrer. The potassium permanganate (6.0 g) was added gradually to the suspension with incredible shaking and observing the rate of addition in order to keep the reaction temperature underneath 20 °C. The ice bath was then expelled, and the blend was mixed overnight at 35 °C until it wound up plainly pale caramel solution. After getting the pale paste, 100 mL water was gradually added to dilute the paste under consistent agitation. The reaction temperature was quickly increased with high effervescence, and the shading changed to darker brown color. After 2 h of vigorous stirring the solution was at long last treated with 30% H_2_O_2_ and the reaction ends up with the presence of brilliant yellow shading. Further, the mixture was flushed a few times with 5% HCl taken after by washing with distilled water till neutralization. After filtration and drying under vacuum at 50 °C temperature, the graphene oxide (GO) was obtained as a gray powder.

#### Sulphonation of graphene oxide

Sulphonation of GO powder as prepared above was done by dispersing 30 mg of GO powder in 5 mL 0.06 M sulfanilic acid solution at 70 °C. To the dispersion, 1 mL of 0.006 M NaNO_3_ was included dropwise and left overnight under steady unsettling. At long last, the resultant dark black sulfonated graphene was isolated by centrifugation, washed with distilled water till neutralization and dried at room temperature^43^.

#### Synthesis of PIn-Au-SGO nanocomposite

Synthesis of PIn-Au-SGO nanocomposite was completed through *in situ* polymerization strategy. At first, indole monomer solution was set up by dissolving 100 mg of indole monomer in 10 mL of dichloromethane. A 2 mL SGO suspension (2 mg mL^−1^) in distilled water was blended with a HAuCl_4_ solution prepared in 0.5 M HCl and continued mixing for 1 h at magnetic stirrer. Further, this solution was gradually added to the indole solution keeping the indole monomer to HAuCl_4_ molar proportion as 1:1 bringing about the development of two particular layers of the separate solutions. The solution was left undisturbed for 24 h for finish polymerization. After polymerization, a greenish dark PIn-Au-SGO composite was obtained, which was isolated and cleaned with distilled water taken after by 0.2 M HCl. At long last, the resultant PIn-Au-SGO nanocomposite was vacuum-dried at 45 °C and saved for further studies.

#### Preparation of PIn-Au-SGO nanocomposites dispersion

The PIn-Au-SGO nanocomposites dispersion was set up by dissolving 4 mg of the synthesized material in 1 mL DMF solvent. The mixture was then ultrasonicated for 30 min. The execution of scattering was measured by a UV–vis spectrophotometer and absorption spectrum between 300–700 nm was recorded.

#### Electrode preparation

A 3 mm diameter glassy carbon (GC) electrode was cleaned to a mirror finish with emery paper and alumina (Al_2_O_3)_ slurry (1.0 and 0.3 mm), utilizing a velvet cushion. The electrode was then ultrasonically cleaned in toluene for 15 minutes and permitted to dry with high virtue nitrogen (N_2_) stream. A 6 µl of this suspension PIn-Au-SGO (as above prepared) was specifically cast on the GC electrode surface, trailed by solvent vanishing at room temperature. Further, a 6 µl of ferritin was drop cast on the dried PIn-Au-SGO composite modified GC electrode and left to dry at room temperature for 1 h. In the wake of drying of the modified electrode, a 10 mg mL^−1^ of the enzyme glucose oxidase (GOx) prepared in phosphate buffer saline (PBS) of pH 5.0 was immobilized. Similar casting and drying technique was used to immobilize 8 µl of GOx on the dried PIn-Au-SGO/Frt biocomposite electrode. At long last, a 2 µl of a 2% glutaraldehyde solution was drop thrown on the modified GC electrode to crosslink the PIn-Au-SGO/Frt/GOx biocomposite solidly and left to dry for a 30 min. The electrode was plunged in deionized water for a time of 2 min to release any non-bound components followed by drying at room temperature and was put in refrigerator until the estimations were taken. The graphical representation of the layer-by-layer immobilization of biomolecules and their electrostatic association on the outlined bioanode appears in Fig. 1.

#### Characterization

As synthesized PIn-Au-SGO nanocomposite was analyzed by electron microscopy (SEM), Fourier transform infrared spectroscopy (FTIR), cyclic voltammetry (CV), linear sweep voltammetry (LSV) and impedance spectroscopy (EIS). Every single electrochemical estimation was performed utilizing a PC controlled Potentiostat/Galvanostat (302 N Autolab, Switzerland) in an ordinary three electrode framework which incorporates a working anode of PIn-Au-SGO/Frt/GOx modified glassy carbon (GC) (Metrohm 6.1204.300), biocomposite electrode, an Ag/AgCl reference electrode and a platinum wire counter electrode. Preceding experimentation, the anode was cleaned with S15H ultrasonic cleaner (Elmasonic, Germany).

## Results and Discussion

### Structural study

**Figure 2:**
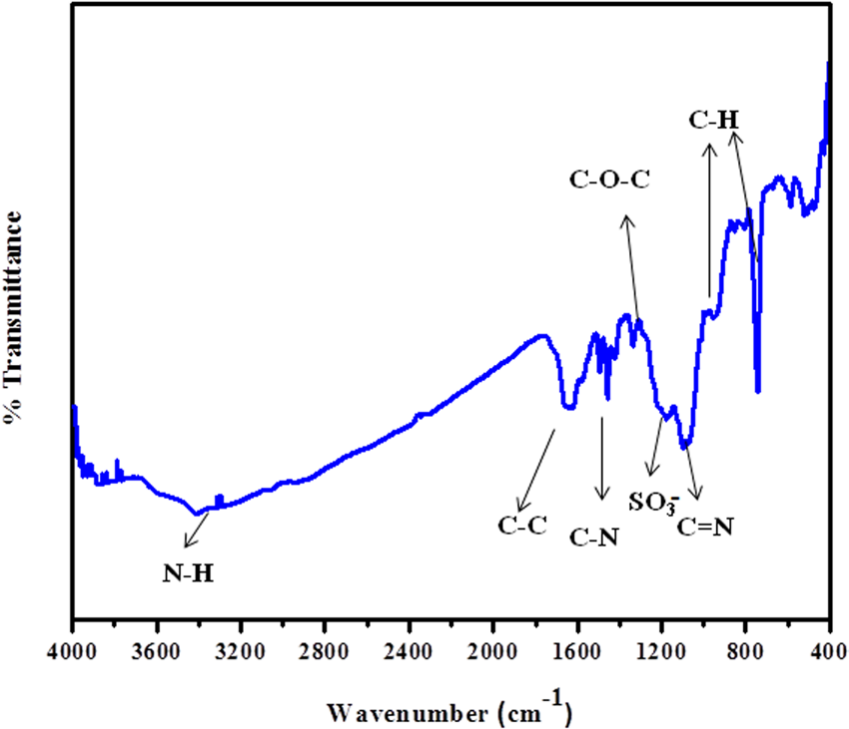
FTIR spectrum of PIn-Au-SGO nanocomposite.

The diverse functionalities of PIn-Au-SGO nanocomposite are observed by FTIR spectroscopic investigation as appeared in Fig. 2. The FTIR spectrum of the as-prepared composite demonstrates the diverse functional groups present in the PIn-Au-SGO nanocomposite. The peak at 3413.92 cm^−1^ is relegated to N-H stretching vibration while the peak at 1098 cm^−1^ relates to the stretching vibration of C = N, 1456 cm^−1^ (ν C-N), C-C at 1652.72 cm^−1^, stretching vibration of C-O-C at around 1378 cm^−1^ and the peak at 1181 cm^−1^ affirms the nearness of sulphonic acid groups while the peaks at 1008 cm^−1^ and at 740 cm^−1^correspond to the in-plane and out-of-plane distortions for C-H bond, individually. The presence of all the peaks of PIn and SGO in the PIn-Au-SGO nanocomposite epitomizes the fruitful association of polyindole with sulfonated graphene oxide^60^. However, the shift in the absorption peaks towards longer wavenumber and additionally variation in the peak intensities is ascribed to the association (coordination bond and electrostatic interaction) between the functional groups of PIn, SGO and gold particles^61,62^.

### Morphological study

**Figure 3:**
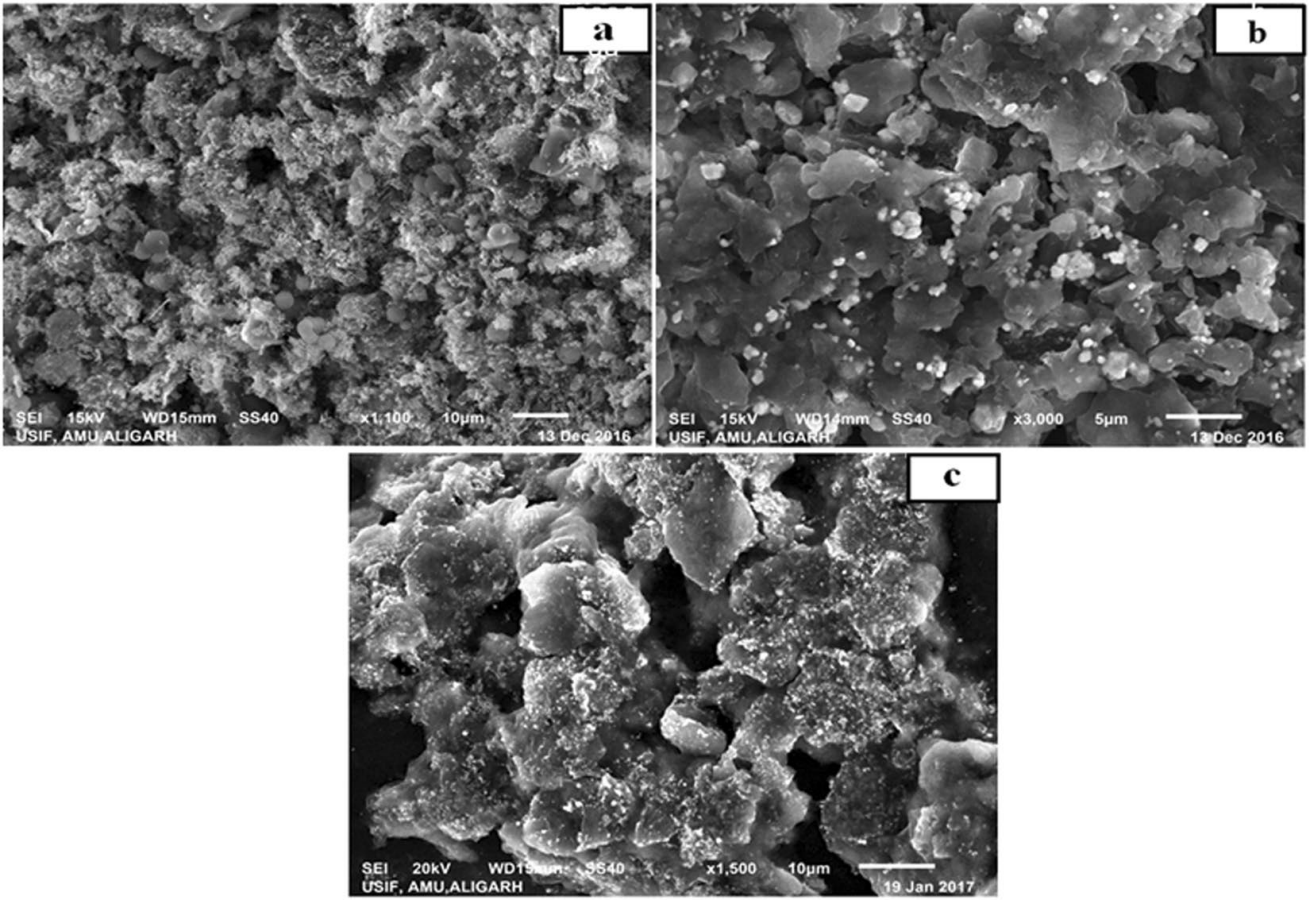
SEM micrographs of (**a**) PIn-Au-SGO nanocomposite, (**b**) GC/PIn-Au-SGO/Frt and (**c**) GC/PIn-Au-SGO/Frt/GOx bioanode.

The morphological surfaces of the PIn-Au-SGO nanocomposite, PIn-Au-SGO/Frt, and GOx immobilized PIn-Au-SGO/Frt biocomposite were explored by SEM as demonstrated in Fig. 3. The composite demonstrates the joining of globular shaped gold nanoparticles with PIn grid in the pores of SGO. Usually, the estimation of the molecular size of reduced gold is found troublesome attributable to the uniform distribution of these metals into the PIn network alongside SGO (Fig. 3a). From picture 3b, a generous change in the surface morphology can be seen because of the deposition of ferritin mediator which was utilized as an electron transfer mediator. This exemplifies that the mediator was evenly adsorbed on the surface of the PIn-Au-SGO nanocomposite. While Fig. 3c manifests the proper immobilization of GOx on the ferritin modified biocomposite wherein the GOx acted as a biocatalyst.

### Electrochemical studies

**Figure 4:**
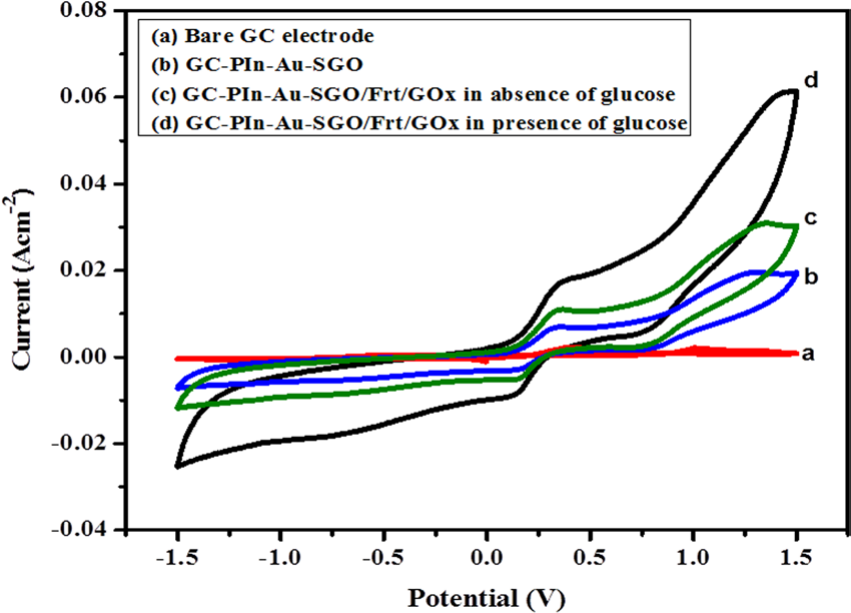
CVs of (**a**) Bare GC electrode, (**b**) GC/PIn-Au-SGO, (**c**) GC/PIn-Au-SGO/Frt/GOx without glucose, (**d**) GC/PIn-Au-SGO/Frt/GOx in presence of glucose in 0.1 M K_4_Fe(CN)_6_ solution at room temperature at scan rate of 100 mVs^−1^.

The electrochemical investigations demonstrated that the modified GC/PIn-Au-SGO/Frt/GOx biocomposite anode displays good electrocatalytic activity toward glucose oxidation due to a synergic effect between PIn, Au, and SGO. The outcome demonstrated the likelihood to utilize this as an anode in the glucose based biofuel cells. The electrocatalytic execution of the PIn-Au-SGO composite modified anode was examined in 0.1 M K_4_Fe(CN)_6_ at 100 mVs^−1^ by cyclic voltammetry to check the execution of the as-orchestrated nanocomposite as a novel network for the viable immobilization of GOx enzyme and to realize the mediated electron transfer utilizing ferritin (up to 4500 iron atoms) as mediator, alongside the required electrical communication of generated electrons from GOx surface to the GC electrode. The outcomes observed are shown in Fig. 4. It is evident from the figure that the uncovered GC electrode produced no catalytic turnover while the GC electrode modified with PIn-Au-SGO demonstrated impressive electrocatalytic oxidation-reduction response with the era of oxidation current of 6.93 mA cm^−2^. This suggests the polymer lattice of polyindole having an electron rich moiety serves as an excellent conducting network for the dispersion of nanometallics (here Au nanoparticles). This is considered the account of good stability and interaction of the heteroatom during redox execution and in addition, capacity to hold nanoclusters within the framework^17,63^. Additionally, the encapsulation of gold nanoclusters within the polyindole network as well as the sulfonation of graphene oxide improves the overall performance of the resulting composite by increasing the electrical and ionic conductivities of the composite altogether. This is expected due to the synergism operating between the electron rich center of polymers, metal nanoparticles and the layered structure of graphene oxide^27,28,64^.

The biocatalytic action of the modified GC/PIn-Au-SGO/Frt connected GOx was electrochemically portrayed by further looking at the response result of the glucose oxidation as appeared in Fig. 4c and 4d. The figure displaying the cyclic voltammograms of the GC/PIn-Au-SGO/Frt/GOx anode both in the presence and absence of 50 mM glucose in 0.1 M K_4_Fe(CN)_6_. Without glucose, a semi-reversible system was seen alongside some improvement in the oxidation current (10.7 mAcm^−2^) because of the addition of Frt protein which suggest that this protein acted as redox mediator and enhanced the electron transfer between the dynamic sites of GOx and the GC electrode while a clear increment in the biocatalytic current up to 17.8 mAcm^−2^ accompanied with an anodic peak was seen in presence of glucose inferring that the GOx is covalently connected to the modified glassy carbon electrode kept up its biocatalytic activity^65^.

The ascent in the oxidation current credits to the intervened mediated electron transfer by ferritin from the profoundly embedded dynamic destinations of the enzyme by moving between the redox dynamic sites of GOx and the electrode-electrolyte interface and the electrical correspondence being given by the adsorbed conducting PIn-Au-SGO nanocomposite that improves the proficiency of the electron transfer of the biocomposite modified anode. Along these lines, ferritin decreases the electron transfer distance by entering the clefts of enzyme and transport between the redox sites of enzyme catalyst GOx and electrode interface. This redox activity clearly affirms the immobilization of GOx in the PIn-Au-SGO matrix and this obviously recommends the outcomes acquired are in great concurrence with the approach of building up a glucose compatible bioelectrode for the application of scaled down implantable gadgets.

**Figure 5:**
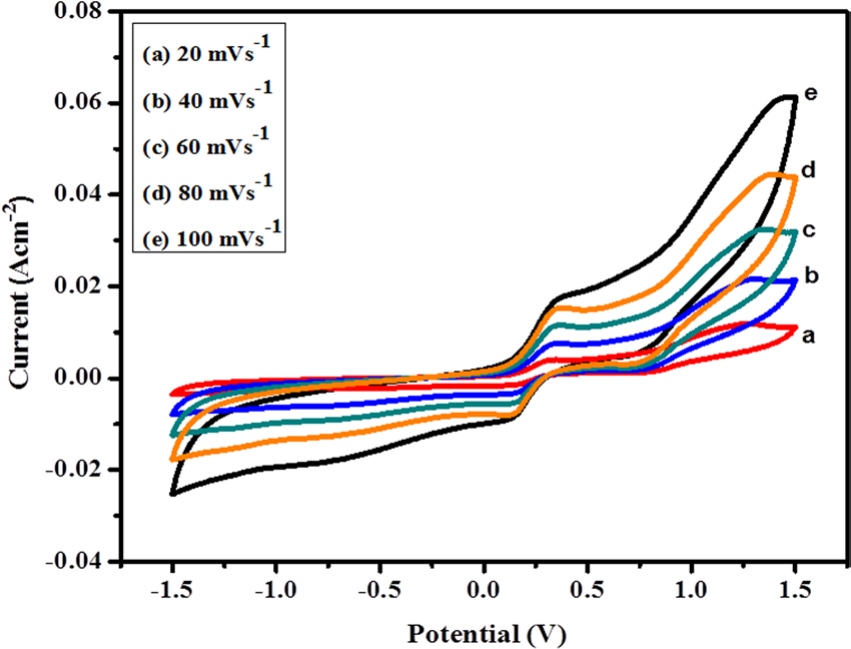
CVs of PIn-Au-SGO/Frt/GOx biocomposite modified electrode at scan rates ranging from 20–100 mVs^−1^.

Keeping in mind the end goal to check the impact of sweep rate on the electrocatalytic execution of GC/PIn-Au-SGO/Frt/GOx toward glucose oxidation, CVs were again performed in 0.1 M K_4_Fe(CN)_6_ at various scan rates shifting from (20–100 mVs^−1^) as appeared in Fig. 5. The outcomes uncover that with an increase in the sweep rate, the oxidation peak current enhanced slowly, which shows the high electrocatalytic nature and semi-reversible redox behavior of the bioanode. The anodic and cathodic peak potentials were additionally observed to be practically autonomous of the scan rates assignable to the faster charge transfer by the ferritin redox mediator.

**Figure 6:**
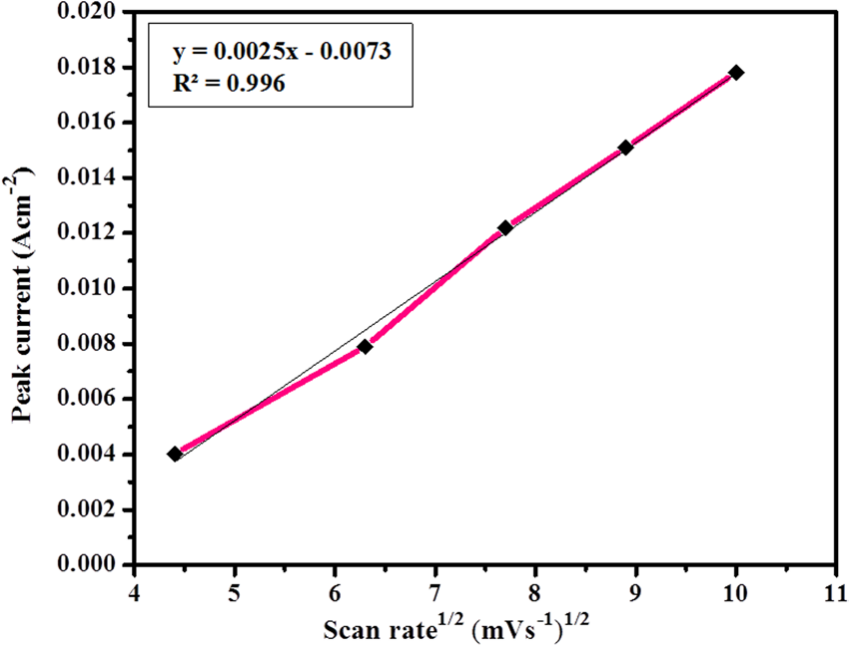
Peak current versus square root of scan rate curve of PIn-Au-SGO/Frt/GOx biocomposite modified electrode.

To affirm the consistency of sweep rate of CV for biocomposite anode, a redox peak current versus square root of scan rate chart was plotted as appeared in Fig. 6. The plot uncovers a linear proportionality relationship between the anodic peak current densities and the square root of the scan rates at fixed glucose concentration (50 mM), recommending that the glucose oxidation by this GC/PIn-Au-SGO/Frt/GOx biocomposite modified electrode is as yet ruled by a diffusion controlled process^66,67^. Thus, it can be concluded that the scan rates have a significant influence on catalytic current.

**Figure 7:**
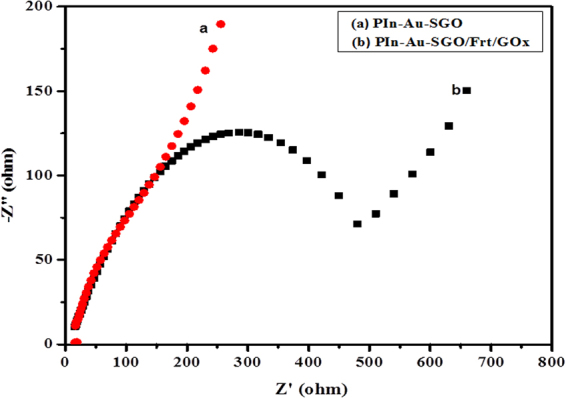
Electrochemical impedance spectra of (**a**) GC-PIn-Au-SGO, (**b**) GC-PIn-Au-SGO/Frt/GOx in 0.1 M K_4_Fe(CN)_6_ solution at 100 mVs^−1^ scan rate.

EIS studies were done to additionally affirm the diffusion controlled phenomena of the GC-PIn-Au-SGO/Frt/GOx bioanode. EIS is an effective technique for analyzing the interfacial aspect of the developed electrodes. Figure 7 displays the typical Nyquist plots of the PIn-Au-SGO and PIn-Au-SGO/Frt/GOx modified GC electrode in 0.1 M K_4_Fe(CN)_6_ as the redox probe. In the impedance estimations, the generation of a straight line is suggestive of a diffusion controlled phenomena whereas the distance across of the crescent part means the resistance to electron transfer (Ret) that manages the charge transfer kinetics of the redox probe at the electrode electrolyte-interface^68,69^.

**Figure 8:**
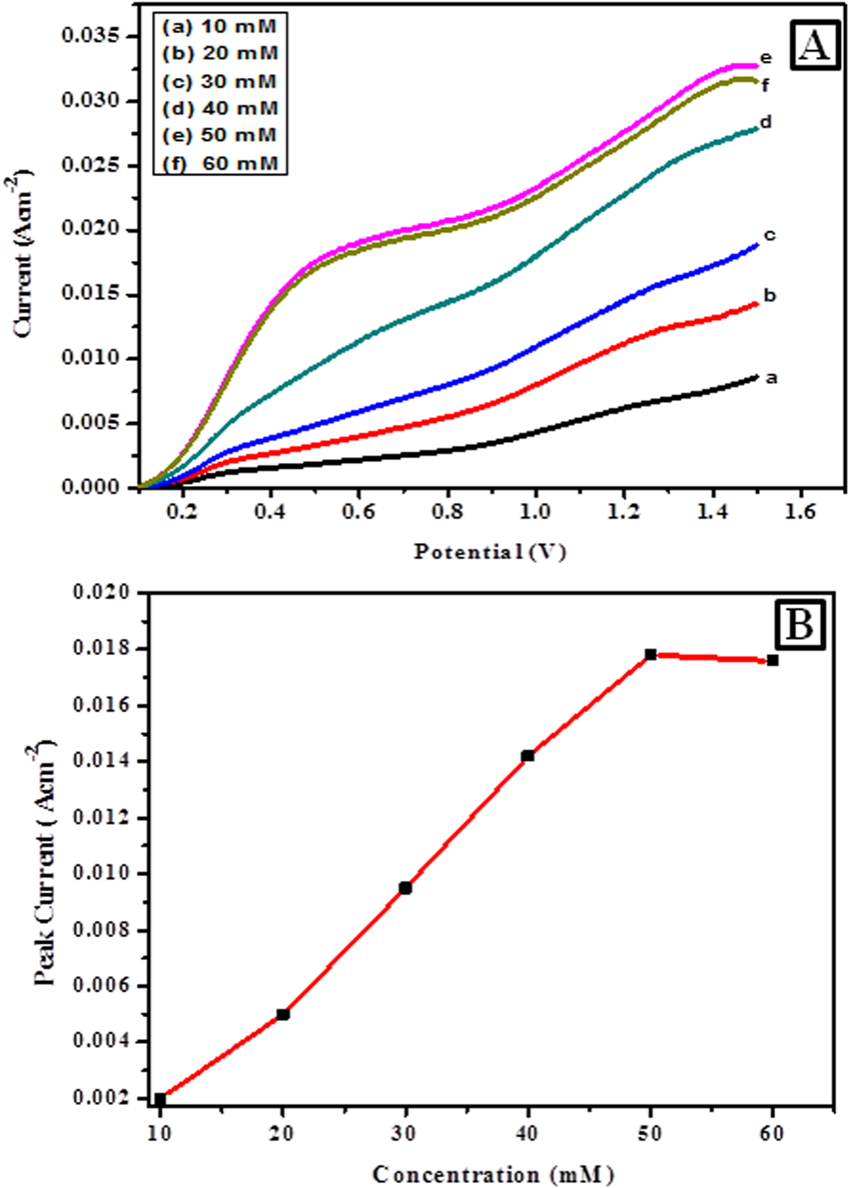
(**A**) LSVs of PIn-Au-SGO/Frt/GOx bioanode in 0.1 M K_4_Fe(CN)_6_ containing different concentrations of glucose varying from (10–60) mM at room temperature with a potential sweep rate of 100 mVs^−1^ and (**B**) the calibration curve corresponding to the electrocatalytic current against the variable concentration of glucose.

It is apparent from the results of Fig. 7 that the electrochemical reaction happening on both PIn-Au-SGO and GOx immobilized PIn-Au-SGO/Frt modified GC electrodes are driven by diffusion controlled pathway and the higher slant of the GC-PIn-Au-SGO curve towards the −Z″ axis clearly displays the higher charge aggregation close to the electrode surface. The insignificantly small semi-circular part of the curve is not shown in the figure for the sake of simplicity. Despite what might be expected, the curve of GC-PIn-Au-SGO/Frt/GOx moderately produced a large diameter semicircle recommending the fruitful immobilization of GOx into the framework of PIn-Au-SGO nanocomposite. In this manner, in the light of the perceptions produced using the EIS results, it can be inferred that the synthesized composite has excellent electrocatalytic properties as a result of the synergistic impact of polyindole, gold and sulfonated graphene oxide including a major contribution by the active gold nanoparticles, however, the significant ascent in the Ret value in case of GOx immobilized electrode is attributed to the polypeptide backbone of the protein of the covalently bound GOx enzyme to the biocomposite modified GC electrode^70,71^. Figure 8A demonstrates LSV curves of the modified PIn-Au-SGO/Frt/GOx bioelectrode with a specific end goal to explore its electrocatalytic oxidation as a function of glucose concentration. The oxidation current response was found to increase directly with an increase in the glucose concentration between 10-60 mM. The observed linear response of biocatalytic current up to 50 mM glucose strongly recommends that the successful enzyme-catalyzed oxidation of glucose happened on the GC electrode modified by the PIn-Au-SGO/Frt/GOx beyond which the current gets stabilized. This leads to draw a reasonable conclusion that the reaction predominantly obeyed a saturation kinetics heading to attain a constant value of the oxidation current that eventually gets saturated with respect to further increase in glucose concentration^72^. The respective calibration for the effect of glucose concentration on the current density generated by the concerned bioanode was drawn as shown in (Fig. 8B). The results mention that a saturation current density of 17.8 mA cm^−2^ was attained at a scan rate of 100 mVs^−1^ for the bioelectrocatalytic oxidation of 50 mM glucose in 0.1 M K_4_Fe(CN)_6_ as the electrochemical marker. Hence, it becomes quite transparent that the proposed bioanode efficiently carried out the electrocatalytic oxidation of glucose via the electron transfer mechanism as appeared in Fig. 1.

It is reasonably considered that the effective immobilization of GOx results in from the covalent binding of GOx with modifiers like sulfonated graphene oxide due to the chemical interaction between the influential COO^-^ groups of SGO and free NH_2_ functionalities of GOx^73^. This covalent bonding keeps the GOx firmly intact with the PIn-Au-SGO nanocomposite suggesting the high stability of the bioanode which is one of the chief factors to check the feasibility of a catalyst to realize its practical implementation. The lifetime of the concerned bioelectrode when stored at 4 °C was estimated to be 53 days (approx). Thus, all the results and excellent stability of the PIn-Au-SGO modified glucose bioanode envisage its dynamic applications in the development of high-performance enzymatic BFCs as well as glucose biosensors. The Brown-Anson model has been applied to determine the surface concentration of PIn-Au-SGO/Frt/GOx deposited on the glassy carbon electrode^74^.

$$Ip=({n}^{2}{F}^{2}{I}^{\ast }Av)/4RT$$

I_p_ symbolizes the anodic peak current, n denotes the number of electrons transferred (here, n = 2),

F is the Faraday constant, I^∗^ stands for the surface concentration, A is the surface area of the electrode, *v*is the scan rate, R is the gas constant, and T is the absolute temperature in Kelvin. The surface concentration of the redox species in PIn-Au-SGO/Frt/GOx bioanode was evaluated to be 2.35 × 10^−9^ mol cm^−2^. A considerably large value of the surface concentration of redox species gives a clear indication of the more active sites of redox centers located in the enzyme being immobilized on the composite matrix that leads to the conclusion that the developed bioanode is potentially active towards diversified electrochemical applications.

## Conclusion

An efficient and cost effective PIn-Au-SGO nanocomposite was successfully synthesized possessing excellent electrocatalytic activity to be utilized as a bioanode (GC/PIn-Au-SGO/Frt/GOx) for the enzymatic glucose biofuel cell application. The composite was characterized by SEM and FTIR methods that embody its fruitful development. The electrochemical examination showed that the concerned PIn-Au-SGO/Frt/GOx modified glassy carbon anode successfully accomplished significant bioelectrocatalytic action toward glucose oxidation utilizing the Frt protein to intercede the electrons and PIn-Au-SGO as electron exchange enhancer^30,33,75^. The accomplished catalytic effectiveness of the PIn-Au-SGO nanocomposite is attainable to the embodiment of gold nanoparticles inside the polyindole network having electron rich moiety and in addition, the sulfonation of graphene oxide enhances the general execution of the subsequent composites by expanding the electrical and ionic conductivities of the composite altogether. This is expected to the synergic impact between the electron rich focal point of polymer, metal nanoparticles and the layered structure of graphene oxide. The biocompatibility and good catalytic efficiency of the subsequent composite not just showing its capability to be utilized as a bioanode in glucose-based biofuel cells, yet can likewise be stretched out to biosensors application.
